# Inner Cell Mass Grade and Earlier Blastulation Are Associated with Pregnancy Outcomes in Euploid Embryos

**DOI:** 10.1007/s43032-025-01971-y

**Published:** 2025-09-11

**Authors:** Yuqi Bian, Sharon H. Zhao, Maxwell Edwin Shramuk, Jacob M. Schauer, Joan Riley, Allison Komorowski, Lia Bernardi

**Affiliations:** 1 Northwestern University Feinberg School of Medicine, 303 E Chicago Ave, Chicago, IL 60611 USA; 2 Biostatistics Collaboration Center, Northwestern University, Chicago, IL USA; 3 Division of Reproductive Endocrinology and Infertility, Northwestern University Feinberg School of Medicine, Chicago, IL USA

**Keywords:** Inner cell mass, Trophectoderm quality, PGT-A, Sex selection

## Abstract

To assess the relationship between blastulation, ICM (inner cell mass) quality, TE (trophectoderm) quality, embryo sex, or embryo sex selection and live birth among euploid embryos. Single-center retrospective cohort analysis of patients who underwent single frozen embryo transfer (FET) following autologous IVF with preimplantation genetic testing for aneuploidy from 2020–2021. Blastocysts underwent trophectoderm biopsy with ICM and TE grading before vitrification. The associations between live birth (LB) and patient/embryo characteristics were assessed with mixed-effect multivariable logistic regression. In 768 euploid FET cycles with available live birth data, 404 cycles (52.6%) resulted in live birth. A significantly lower odds of LB was seen with vitrification on days 6 and 7 compared to day 5 (aOR 0.81, 95% CI 0.70–0.95) and in embryos with lower ICM quality compared to those with quality 1, the highest of three quality grades and indicative of tightly packed cells (aOR 0.75, 0.62–0.90). Embryo sex was known in > 90% of transferred embryos, with 355 (50.8%) female and 344 (49.2%) male. In 161 (21.0%) cycles where embryo sex was used to select embryo for transfer with 84 (52.2%) male and 77 (47.8%) female, embryo sex and embryo sex selection were not significantly associated with the odds of LB. Prioritizing embryos for transfer with better ICM quality and earlier blastulation may provide the highest chance of LB. Trophoblast quality at vitrification, embryo sex, and embryo sex selection were not associated with the odds of LB, but these factors may influence patient and physician decision-making.

**Supplementary Information:**

The online version contains supplementary material available at 10.1007/s43032-025-01971-y.

## Introduction

The goal of an embryo transfer, as the culmination of an in vitro fertilization (IVF) cycle, is to transfer an embryo that has the best chance of leading to a live birth. Embryo morphology and day of blastocyst development have traditionally been used as predictors of implantation and live birth, and thus embryologists use these factors to determine which embryo(s) to preferentially transfer in an effort to achieve the highest likelihood of success [1]. Morphology grading systems have conventionally described the inner cell mass (ICM) and trophectoderm (TE) cells of blastocysts [2]. However, it has been debated which morphology score component has greater strength in predicting implantation rates and pregnancy outcomes [3–5]. Day of blastulation has also been utilized as a predictor of clinical outcomes, although studies have reported varied results concerning implantation rates and pregnancy outcomes between day 5 and day 6 transfers [6–8]. Nevertheless, morphology and day of blastulation remain some of the few non-invasive parameters that can be used when determining how to prioritize which embryo to transfer. Generally, embryos with higher morphologic scores and those that blastulate earlier are transferred preferentially to optimize pregnancy outcomes after embryo transfer [9].

Preimplantation genetic testing (PGT) is commonly used in conjunction with IVF to enhance the selection of embryos. PGT for aneuploidy (PGT-A) offers information on embryo ploidy and is pursued for a variety of indications, including advanced reproductive age and a history of recurrent pregnancy loss, among other indications [10–12]. One goal of PGT-A is to increase the chance of live birth with a single embryo transfer, and as such, PGT-A as emerged as an embryo selection tool [13, 14]. However, previous literature has arrived at varying conclusions on how and whether non-invasive measures, such as morphology and day of blastulation, can be utilized in addition to PGT-A when selecting which euploid embryo to transfer [15].

When PGT-A is performed in some areas around the world, embryo sex can also be determined if desired by the patient and permitted by the clinic. Although clinics differ in their policies regarding sex selection, embryo sex is another factor that may lead to preferential transfer of one embryo over another for some patients. Some data have revealed sex-related differences in pregnancy outcomes while other data do not demonstrate differences by embryo sex [16–18]. Furthermore, rates of sex selection following PGT-A have been studied, but few studies have examined whether pregnancy outcomes differ when a patient selects an embryo for transfer based on embryo sex [19, 20].

There is currently a paucity of literature investigating the associations between PGT-A, sex selection, morphology, day of blastulation, and IVF cycle outcomes. In this study, we aimed to assess whether day of blastulation, ICM quality, TE quality or embryo sex were associated with live birth following euploid embryo transfer. We also examined if there was an association between live birth and whether an embryo was selected for transfer based on sex.

## Methods

### Inclusion Criteria

This is a single-center retrospective cohort study that analyzed all single frozen embryo transfer (FET) cycles that occurred at Northwestern Medicine within the Division of Reproductive Endocrinology and Infertility between January 2020 and July 2021. All embryos that were transferred were from autologous IVF cycles and had undergone trophectoderm biopsy followed by preimplantation genetic testing for aneuploidy (PGT-A). Only cycles with embryos deemed to be euploid through PGT-A were included in analysis. Data on IVF cycle parameters, patient characteristics, and pregnancy outcomes were collected via electronic medical record review.

### Ethical Approval

This study was reviewed and approved by the Northwestern University Institutional Review Board (STU00217151, 6/27/2022).

### Ovarian Stimulation, Oocyte Retrieval, and Embryo Culture

All patients underwent controlled ovarian stimulation with our institution’s standard protocols. These protocols utilized either a gonadotropin-releasing hormone (GnRH) antagonist, luteal phase leuprolide acetate suppression, or a GnRH agonist flare. Subcutaneous gonadotropins were administered to stimulate follicular development. Ovarian response was monitored via monitoring of serum estradiol level and abdominal or transvaginal ultrasound. Oocyte retrieval was performed 36 h after injection of human chorionic gonadotropin (hCG) and/or a GnRH agonist (leuprolide acetate) for induction of final oocyte maturation. Oocytes were inseminated by either intracytoplasmic sperm injection (ICSI) or conventional insemination. After oocyte retrieval, recombinant human hyaluronidase 40–120 U/mL (Origio) was used to denude cumulus cells to assess nuclear maturity in oocytes that were to undergo ICSI. Embryos were cultured in continuous single culture-NX-complete media (FujiFilm/Irvine Scientific) using the EmbryoScope time lapse system (Vitrolife). Normal fertilization was confirmed on day 1. On day 3, cell stage (6 blastomeres or greater being optimal), as well as fragmentation and symmetry were determined. Embryos received a fragmentation score of 1 (< 10%), 2 (10–25%), or 3 (> 25%) in addition to a symmetry score of 1 (all similarly sized), 2 (1–2 cells uneven in size), or 3 (> 2 cells uneven in size).

### Trophectoderm Biopsy and Vitrification

**Fig. 1:**
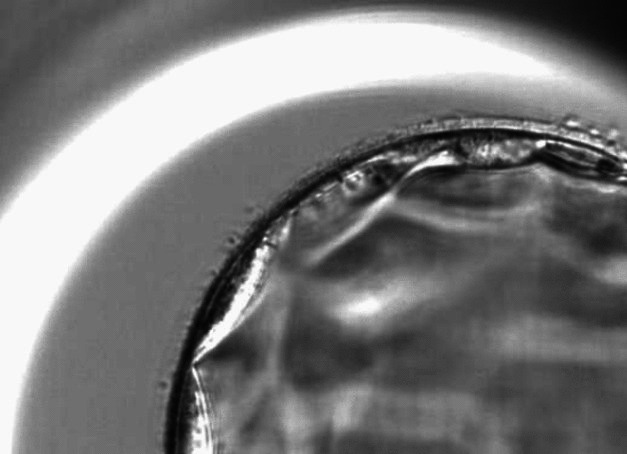
Embryo with inner cell mass (ICM) grade 1 and trophectoderm (TE) grade 1

**Fig. 2:**
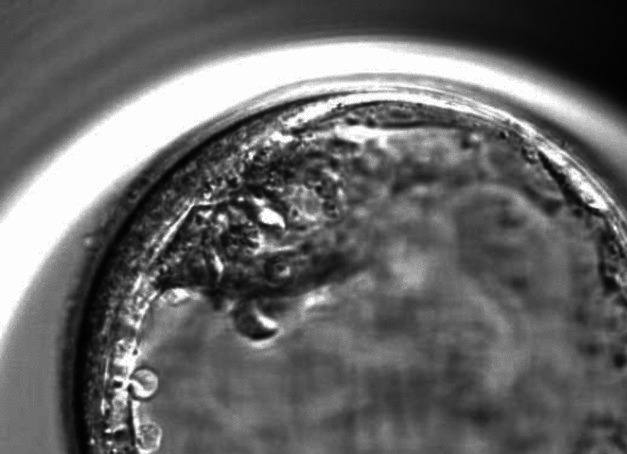
Embryo with inner cell mass (ICM) grade 2 and trophectoderm (TE) grade 2

On day 5, embryo quality was assessed to determine if a trophectoderm biopsy could be performed. Embryos that were not able to be biopsied on day 5 were re-evaluated on day 6, with some evaluated again on day 7 to determine if their quality was sufficient for biopsy. Morphology grades of 1, 2, or 3 were assigned to the ICM and TE, with 1 being the most desirable grade and 3 being least desirable. For ICM, grade 1 indicated a well-formed tightly packed ICM with many cells, with 3 indicating very few cells. For TE, grade 1 indicated many cells forming a cohesive epithelium, while 3 indicated a TE with very few large cells. Grades of 2 for ICM and TE were assigned if morphology was in between what was described for grades 1 and 3 (See Figs. 1 and 2).

Any embryo that appeared high quality by days 6 or 7 had a biopsy performed in which approximately 5–7 trophectoderm cells were removed. After an embryo underwent trophectoderm biopsy, it was immediately cryopreserved by vitrification and stored in liquid nitrogen. Grading was conducted at time of biopsy before vitrification. Assisted hatching was completed at time of biopsy. Each biopsy was sent to one of two clinical laboratories for evaluation, and PGT-A testing was performed using SNP-array or next generation sequencing (NGS). Embryos were subsequently categorized as euploid, aneuploid, or mosaic. The aneuploid thresholds for the two labs are > 80% and > 70%. Clinical outcomes, including pregnancy rate and euploidy rate, are examined by the practitioner completing the biopsy, and are tracked for quality control in the embryology laboratory.

### Embryo Transfer

Patients prepared for frozen embryo transfer (FET) using either a medicated or natural cycle approach. For those who underwent medicate FET cycles, the majority of patients used estradiol (oral, vaginal or patch) initiated at the early follicular phase of a menstrual cycle and began intramuscular and vaginal progesterone once the endometrial lining reached 7 mm. Single frozen transfers were performed on the 6th day of progesterone administration. If pregnancy was confirmed, exogenous estradiol and progesterone were continued until 9 weeks of gestation. For patients undergoing a mild stimulation cycle FET, letrozole was used for ovulation induction; for patients undergoing modified natural cycle FET, ovulation was triggered with hCG. Luteal support was administered per physician preference. The embryology team chose the morphologically best euploid embryo for transfer. Although our clinic does not permit IVF for sex-selection or extraneous IVF cycles to obtain an embryo of a certain sex, patients with embryos of different sexes with similar morphology grades were permitted to view embryo sex when choosing which embryo to preferentially transfer.

### Statistical Analysis

The primary outcome was live birth, defined as a liveborn child born at 24 weeks gestation or later. The odds of a pregnancy resulting in a live birth was modeled through generalized linear mixed effects models (GLMM) using a logit link (i.e., mixed effects logistic regression) with random patient effects. To assess associations between patient/embryo characteristics and live birth, GLMM included fixed effects for variables selected based on statistical significance (p ≤ 0.1) of bivariate relationships (i.e., Fisher’s exact test, Chi-square test, Wilcoxon/t-tests), whether inclusion decreased the Akaike Information Criterion (AIC), and whether they modified the relationship between other variables and the odds of live birth. Variables that met these requirements were blastocyst expansion degree, cleavage stage fragmentation score, patient obesity status, and age at oocyte retrieval. GLMM further included fixed effects for the following clinically relevant variables to analyze their association with live birth: embryo ICM and TE quality at freezing, the day of development on which embryos were vitrified, whether the embryo was chosen based on sex, and embryo sex. An analysis of the variables of ICM quality and vitrification day was then performed to account for a potential confounding relationship. Subgroup analyses stratified on embryo sex were also created for each model. Pairwise group comparisons were conducted for all permutations of ICM quality, vitrification day, and embryo sex, with p-values adjusted using a Bonferroni cutoff of *p* = 0.0018. MICE (m = 5 imputations) version 3.16.0 was used to address missing data [21]. All analyses were performed in R version 4.3.2 and SAS version 9.4 (SAS Institute Cary NC) using the GENMOD procedures. ICM grades 2 and 3, TE grades 2 and 3, and day 3 fragmentation values 2 and 3, vitrification days 6 and 7, and embryo stages of blastocyst and early blastocyst were combined in analysis to maximize statistical power. Of note, the cluster-specific coefficients used in this analysis were also converted to population-average coefficients using the correction by Austin and Merlo (2017). The differences between the coefficients were negligible (< 1%), thus the odds ratios are nearly identical. This means the results of this study’s analyses can be compared to other analyses addressing a similar research question.

## Results

A total of 781 euploid FET cycles across 576 patients were collected and analyzed, with 768 having available data regarding pregnancy outcome. Patient demographics and embryo details for cycles with available pregnancy outcomes are included in Table 1. Of all FET cycles analyzed, almost two thirds used embryos vitrified on day 5. In analysis of quality, more than 80% of embryos were quality 1 for ICM while nearly two thirds had TE quality 1. Over 95% of embryos were fertilized via ICSI (Table 1).

**Table 1:** Patient demographics and embryo details of euploid frozen embryo transfer cycles

	Overall (*n* = 768 cycles)	Live Birth (*n* = 404 cycles)	No Live Birth (*n* = 364 cycles)
**Age at oocyte retrieval,** years (mean ± SD [range])	35.5 ± 3.4 [25.0–45.0]	35.6 ± 3.5 [25.0–44.0]	35.4 ± 3.4 [27.0–45.0]
**Age at embryo transfer,** years (mean ± SD [range])	36.3 ± 3.6 [25.0–47.0]	36.3 ± 3.6 [26.0–44.0]	36.3 ± 3.7 [25.0–47.0]
**Body mass index,** kg/m^2^ (median [IQR])	24.0 [21.6–28.5]	23.8 [21.6–27.7]	24.3 [21.5–29.2]
**Meeting obesity criteria** (BMI > 30 kg/m^2^)	139 (18.1%)	57 (14.1%)	82 (22.6%)
**Fertilization method utilized**			
Intracytoplasmic sperm injection	695 (95.6%)	376 (95.9%)	319 (95.2%)
Conventional insemination	32 (4.4%)	16 (4.1%)	16 (4.8%)
**% fragmentation at cleavage stage**^**a**^			
1	570 (79.4%)	318 (82.2%)	252 (76.1%)
2	135 (18.8%)	63 (16.3%)	72 (21.8%)
3	13 (1.8%)	6 (1.6%)	7 (2.1%)
**Symmetry at cleavage stage**^**a**^			
1	439 (61.1%)	238 (61.5%)	201 (60.7%)
2	273 (38.0%)	146 (37.7%)	127 (38.4%)
3	6 (0.8%)	3 (0.8%)	3 (0.9%)
**Day of embryo vitrification**^**a**^			
Day 5	483 (63.0%)	276 (68.3%)	207 (57.0%)
Day 6	282 (36.8%)	128 (31.7%)	154 (42.4%)
Day 7	2 (0.3%)	0 (0.0%)	2 (0.6%)
**Quality of inner cell mass at vitrification**^**a**^			
1	629 (82.0%)	349 (86.4%)	280 (77.1%)
2	137 (17.9%)	55 (13.6%)	82 (22.6%)
3	1 (0.1%)	0 (0.0%)	1 (0.3%)
**Quality of trophoblast at vitrification**^**a**^			
1	488 (63.6%)	263 (65.1%)	225 (62.0%)
2	278 (36.3%)	141 (34.9%)	137 (37.7%)
3	1 (0.1%)	0 (0.0%)	1 (0.3%)
**Sex of embryo**^**a**^			
Female	355 (50.8%)	213 (53.0%)	142 (47.8%)
Male	344 (49.2%)	189 (47.0%)	155 (52.2%)

Unit of observation is one cycle. Multiple cycles may belong to a single individual. Except for age and BMI, all other variables are categorical and use N (%)

^a^Data for these parameters were not available for all 768 cycles

Embryo sex was known from PGT-A reports in over 90% of the transferred embryos. Of those with known embryo sex, there were 355 (50.8%) female embryos and 344 (49.2%) male embryos transferred. In 161 (21.0%) cycles, embryo sex preference was used to guide which embryo to transfer. For these cycles, 84 (52.2%) of embryos were male while 77 (47.8%) were female.

There were 404 FET cycles that resulted in live birth (52.6%). In GLMM analyses, while controlling for other morphological factors, embryos biopsied and vitrified on days 6 and 7 demonstrated significantly lower odds of live birth than those vitrified on day 5 (aOR 0.81, 95% CI 0.70–0.95) (Table 2). Lower quality of ICM at vitrification was also associated with significantly lower odds of LB as compared to those with quality 1 ICM (aOR 0.75, 95% CI 0.62–0.90). The interaction term between ICM quality and vitrification day was not statistically significant (aOR 1.03, 95% CI 0.85–1.26), indicating that neither variable moderates the other’s relationship with live birth. Quality of TE at vitrification was not significantly associated with odds of live birth (aOR 0.93, 0.80–1.08).

**Table 2:** Association between morphologic measures and live birth

Parameter	Variable of Interest	Adjusted Odds Ratio	95% Confidence Interval	*p*-value
Day of embryo vitrification	Day 6 or 7	0.81	0.70–0.95	**0.0088**
Quality of inner cell mass at vitrification	Quality 2 or 3	0.75	0.62–0.90	**0.0026**
Quality of trophoblast at vitrification	Quality 2 or 3	0.93	0.80–1.08	0.3638

Multivariable logistic regression after controlling for obesity status, age at retrieval, blastocyst expansion at thaw, and cleavage stage fragmentation quality. *p*<0.05 is considered significant and has been bolded

Table 3 demonstrates live birth rates by embryo sex, quality of ICM at vitrification and day of vitrification. As shown in Table 4, neither embryo sex itself, nor whether embryo sex was used to guide which embryo to transfer, exhibited a statistically significant association with odds of live birth. Among male embryos, only day of vitrification was statistically significantly associated with live birth, with those vitrified on days 6 and 7 having lower odds of live birth than those vitrified on day 5 (aOR 0.77, 95% CI 0.61–0.97). For female embryos, only quality of ICM was significantly associated with live birth (aOR 0.66, 95% CI 0.51–0.87). All other variables were not significantly associated with live birth in analyses stratified by sex.

**Table 3:** Live birth rates by embryo sex, quality of inner cell mass at vitrification and day of embryo vitrification

Day 5, ICM 1		Day 5, ICM 2		Day 6, ICM 1		Day 6, ICM 2	
Female	60.9%	Female	42.3%	Female	54.9%	Female	36.0%
Male	56.4%	Male	44.9%	Male	41.0%	Male	39.3%

**Table 4:** Association between morphologic measures and live birth by embryo sex

	Variable of Interest	Adjusted Odds Ratio	95% Confidence Interval	*p*-value
**Embryo sex**	Female	1.08	0.93–1.27	0.3163
**Method of embryo selection**	Based on embryo sex	1.01	0.85–1.21	0.8845
**Male embryos**				
Day of embryo vitrification	Days 6 and 7	0.77	0.61–0.97	**0.0236**
Quality of inner cell mass at vitrification	Quality 2 and 3	0.83	0.63–1.10	0.1893
Quality of trophoblast at vitrification	Quality 2 and 3	0.99	0.79–1.24	0.9282
**Female embryos**				
Day of embryo vitrification	Days 6 and 7	0.85	0.68–1.06	0.1429
Quality of inner cell mass at vitrification	Quality 2 and 3	0.66	0.51–0.87	**0.0030**
Quality of trophoblast at vitrification	Quality 2 and 3	0.87	0.70–1.08	0.2079

Multivariable logistic regression after controlling for obesity status, age at retrieval, embryo stage at thaw, and day 3 fragmentation quality. *p*<0.05 is considered significant and has been bolded

Supplementary Table 1 shows the results of pairwise group comparisons between patient groups with all permutations of the variables of vitrification day, quality of ICM at vitrification, and embryo sex. Notably, female embryos vitrified on day 6 with quality 1 ICM had significantly higher odds of live birth compared with both male (OR 1.23, *P* < 0.0001) and female embryos (OR 1.36, *P* < 0.0001) vitrified on day 5 with quality 2 ICM. In addition, male embryos vitrified on day 6 with quality 1 ICM were associated with higher odds of live birth than male embryos vitrified on day 5 with quality 2 ICM (OR 1.11, *P* = 0.0003), while male embryos vitrified on day 6 with quality 1 ICM did not have a significantly higher odds of live birth when compared to female embryos vitrified on day 5 with ICM quality 2 (OR 1.23, *P* = 0.0066). In addition, female embryos vitrified on day 6 with quality 1 ICM had significantly higher odds of live birth compared to male embryos vitrified on day 6 with the same quality ICM (OR 1.11, *P* < 0.0001).

## Discussion

We found that both day of vitrification and quality of the inner cell mass at vitrification were significantly associated with odds of live birth in euploid embryos. In contrast, the quality of the trophoblast at vitrification, the sex of the embryo, and whether an embryo was selected for transfer based on sex were not associated with odds of live birth.

Prior studies of untested embryos demonstrated that embryos that blastulate and are vitrified earlier have more favorable pregnancy outcomes. When frozen embryos transferred after vitrification on day 7 were compared with those that were vitrified by days 5 or 6, day 7 embryos had lower rates of implantation, clinical pregnancy, and live birth [22, 23]. Within studies that compared untested embryos that were vitrified on days 5 versus 6, live birth rates were also higher in the embryos that were vitrified earlier for both fresh and frozen cycles [6, 24, 25]. Additional studies have evaluated live birth outcomes in euploid embryos with varied results. Irani et al. and Boynukalin et al. found that among single frozen euploid transfers, embryos vitrified on day 6 were associated with lower live birth rate than those vitrified on day 5, which is consistent with the results of our study [26, 27]. However, Shear et al., Ji et al. and Gonzalez et al. did not find a significant association between day of vitrification (day 5 vs day 6) and live birth in euploid embryos [28–30]. This is in concordance with results reported by Capalbo et al., who found that blastocyst developmental rate was not predictive of implantation rate [31]. Thus, our results are in agreement with some previous studies of both untested and euploid embryos, although there is discordance amongst existing literature. It is possible that these discrepancies are Due to methodological differences. Gonzalez et al. focused their study solely on embryos that underwent PGT-A through NGS, while our study includes PGT-A conducted via both NGS and SNP-array; furthermore, their cohort was of higher maternal age and of smaller sample size than our study. The authors also noted that their initial analysis suggested day 6 euploid embryos had lower live birth rate than day 5 euploid embryos, although this lost significance in the final analysis. The studies led by Ji et al. and Capalbo et al. both had smaller cohort sizes, which may have limited the statistical power of the studies.

Our data also showed an association between live birth and ICM quality, but not TE quality, demonstrating that not all measures of embryo morphology were associated with odds of live birth. These results align with prior studies that have found ICM to be either the only or the most significant morphological predictor of ongoing pregnancy and live birth in euploid embryos following PGT-A [4, 29, 32, 33]. In contrast, other studies have reported the superiority of TE grading in predicting live birth across both untested and euploid embryos. In a study of fresh blastocyst transfers, Ahlstrom et al. found TE quality to be the only independent predictor of live birth when analyzed along with ICM and blastocele expansion degree, although this study included fresh blastocyst transfers with unknown ploidy [34]. In a study of euploid embryos, Rienzi et al. similarly found an association between TE quality and live birth, with ICM remaining statistically insignificant [35]. Yet other studies of euploid embryos have found no significant association between either measure of morphology or live birth [26, 30, 31]. Improved grading standardization and subsequent additional research using more standardized grading would likely help better characterize the role of morphology in predicting outcomes in euploid embryos.

Although the association between pregnancy outcomes and both day of vitrification and morphology have been previously evaluated, few studies have investigated whether one measure should be prioritized over the other when selecting an embryo for transfer. Understanding whether transferring an embryo vitrified on day 5 with less ideal morphology leads to better outcomes than transferring an embryo vitrified on day 6 with better morphology is important for counseling of patients who want the highest chance of a success after a single embryo transfer. In a study of frozen transfers of untested embryos, Shi et al. concluded that day 6 embryos with a better morphology had a higher live birth rate than day 5 embryos with less ideal morphology [36]. Our study similarly found that embryos with higher ICM scores that were vitrified on later days were associated with a higher odds of live birth than embryos with lower ICM quality that were vitrified on day 5 when comparing within both male and female embryos (Supplemental Table 1). However, in another study of only euploid embryos, Gordon et al. reported that day 5 embryos with less ideal morphology had similar ongoing pregnancy and live birth rates compared to day 6 embryos with better morphology, although the former group had higher rates of miscarriage [9].

While many studies have investigated the influence of PGT-A on sex ratio, few have studied the association between embryo sex and pregnancy outcomes among euploid embryos. In a large-scale analysis of births following IVF, Shaia et al. found that the use of PGT increased the chance of having a male infant in comparison to those who did not use PGT even after excluding transfers conducted for sex selection [37]. This finding is corroborated by Kulmann et al. and Zhang et al., who reported a greater proportion of male births following PGT-A in the absence of sex selection [38, 39]. The authors attributed these results to the extended culture of PGT-A favoring male embryo development, as well as potential genetic factors that favor male embryo survival [37, 38]. This observation may also be explained by studies showing that trophectoderm and general morphological scoring were associated with male embryos within both untested and euploid embryos, resulting in more male embryos chosen based on morphologic factors [38, 40–44]. Our study also documented a difference between embryos of different sex when evaluating the association between morphologic measures and live birth. Day of vitrification was the only morphologic feature significantly associated in male embryos, which is a departure from our finding that ICM grade is associated with live birth amongst all embryos. In contrast, only ICM grading was significantly associated with live birth in female embryos. This suggests a possible inherent difference in development and morphology between female and male embryos, as has been reported in previous literature, which may explain why the differing associations with morphology in male and female embryos did not translate to differences in live birth [37, 38]. However, it is difficult to elucidate why these different associations between male and female embryos did not influence pregnancy outcomes, and further investigations with larger sample sizes are needed to better understand the relationship between sex and embryo development, morphology, and pregnancy outcomes after embryo transfer.

An analysis conducted by Bakkensen et al. found that allowing sex selection after PGT-A did not impact the sex ratio of offspring, although the specific association between sex selection and live birth was not investigated [20]. Previous literature has also demonstrated that implantation and live birth rates are similar between male and female euploid embryos, and that untested embryos of both sexes are equally likely to be euploid and develop at similar rates [16, 17]. Similarly, our study found neither embryo sex nor whether an embryo was selected for transfer based on sex to be significantly associated with pregnancy outcomes. This finding is valuable for guiding counseling for parents who may have a preference for which sex embryo to transfer.

The number of embryo transfers evaluated is a strength of this study, which allowed for reasonably precise analyses among different subgroups of our cohort, with most 95% confidence interval for ORs narrower than 0.3–0.4 in width. That said, there are some limitations of this study. We were only able to evaluate outcomes in our single center, which may decrease the generalizability of these results to other clinics. The subjective nature of morphologic grading and the widespread use of the Gardner criteria for morphological grading outside of our center increase the difficulty of extrapolating our results to other facilities [45]. Furthermore, a small number of embryos were biopsied and vitrified at other centers. Additionally, factors such as indication for undergoing PGT-A, type of endometrial preparation, and maternal characteristics outside of age and BMI were not adjusted for in this study. While maternal age is a factor that has been shown to influence embryo development and outcomes, we did not adjust for age in this study because prior studies have shown that pregnancy outcomes in PGT-A-tested embryos are similar by maternal age [46, 47]. Studies showing age-dependent differences in pregnancy outcomes have reported conflicting outcomes. A meta-analysis led by Vitagliano et al. demonstrated an association between maternal age > 35 years and a decrease in the composite outcome of ongoing pregnancy rate and live birth rate, independent of embryo ploidy [48]. Conversely, Harris et al. showed that cumulative live birth was significantly less in those with maternal age < 35 years and more likely in those with age 38–40 years, compared with no PGT-A [49]. However, a subgroup analysis limited to FET found that women ages 35–40 had an increase in live birth in cycles using PGT-A compared to morphology alone, while this relationship was insignificant in those ages < 35 [49]. To determine the clinical significance of these findings and to provide clarity to the conflicting evidence, additional investigation into the relationship between maternal age and pregnancy outcomes in PGT-A embryo transfers is critical. The authors also recognize that blastocoele expansion, which was not explored in this study, is a component of embryo grading. However, it was not included in this study as our center’s embryology lab uses a less conventional grading system that was difficult to standardize at the time of data collection. There are also a low number of ICM and TE grade 3 embryos included in this study Due to the dataset available at our center; to mitigate potential biases, we grouped grade 2 and grade 3 embryos together. Lastly, although all attempts are made for day of vitrification to be determined by an embryo’s eligibility for trophectoderm biopsy, laboratory factors may have contributed to some embryos being biopsied and vitrified on an earlier or later day in our clinic than they would have been in another center.

In conclusion, euploid embryos with better ICM quality and those that reached the blastocyst stage earlier were associated with higher odds of live birth, suggesting that prioritizing embryos for transfer by these parameters may provide patients with the best chance of live birth.

## Supplementary Information

Below is the link to the electronic supplementary material.

Supplementary file 1 (DOCX 16.8 KB) Pairwise comparisons across embryo sex, quality of inner cell mass at vitrification, and day of embryo vitrification

## Data Availability

Available upon request.
