# Supplementary material for: Inner Cell Mass Grade and Earlier Blastulation Are Associated with Pregnancy Outcomes in Euploid Embryos
Source: Reprod Sci. 2025 Sep 11;32(10):3410–8. doi: 10.1007/s43032-025-01971-y (PMC12546501; doi:10.1007/s43032-025-01971-y)
Supplement: Supplementary file 1 — Pairwise comparisons across embryo sex, quality of inner cell mass at vitrification, and day of embryo vitrification [file 43032_2025_1971_MOESM1_ESM.docx]

**Inner cell mass grade and earlier blastulation are associated with pregnancy outcomes in euploid embryos**

**Supplementary Table 1.** Pairwise comparisons across embryo sex, quality of inner cell mass at vitrification, and day of embryo vitrification

| **Embryo parameters** | **Embryo parameters (reference)** | **Odds Ratio** | **Standard Deviation** | **p-value** |
| --- | --- | --- | --- | --- |
| **Male, ICM 2, Day 5** | **Male, ICM 1, Day 5** | **0.78** | **0.01** | **<0.0001** |
| Female, ICM 1, Day 5 | Male, ICM 1, Day 5 | 1.03 | 0.01 | 0.0030 |
| **Female, ICM 2, Day 5** | **Male, ICM 1, Day 5** | **0.70** | **0.02** | **<0.0001** |
| **Male, ICM 1, Day 6** | **Male, ICM 1, Day 5** | **0.87** | **0.01** | **<0.0001** |
| **Male, ICM 2, Day 6** | **Male, ICM 1, Day 5** | **0.70** | **0.02** | **<0.0001** |
| Female, ICM 1, Day 6 | Male, ICM 1, Day 5 | 0.96 | 0.02 | 0.0464 |
| **Female, ICM 2, Day 6** | **Male, ICM 1, Day 5** | **0.68** | **0.03** | **<0.0001** |
| **Female, ICM 1, Day 5** | **Male, ICM 2, Day 5** | **0.76** | **0.04** | **<0.0001** |
| **Female, ICM 2, Day 5** | **Male, ICM 2, Day 5** | **0.90** | **0.01** | **<0.0001** |
| **Male, ICM 1, Day 6** | **Male, ICM 2, Day 5** | **1.11** | **0.03** | **0.0003** |
| **Male, ICM 2, Day 6** | **Male, ICM 2, Day 5** | **0.90** | **0.03** | **0.0002** |
| **Female, ICM 1, Day 6** | **Male, ICM 2, Day 5** | **1.23** | **0.06** | **<0.0001** |
| Female, ICM 2, Day 6 | Male, ICM 2, Day 5 | 0.88 | 0.05 | 0.0104 |
| **Female, ICM 2, Day 5** | **Female, ICM 1, Day 5** | **0.68** | **0.01** | **<0.0001** |
| **Male, ICM 1, Day 6** | **Female, ICM 1, Day 5** | **0.89** | **0.02** | **<0.0001** |
| **Male, ICM 2, Day 6** | **Female, ICM 1, Day 5** | **0.68** | **0.02** | **<0.0001** |
| **Female, ICM 1, Day 6** | **Female, ICM 1, Day 5** | **0.93** | **0.01** | **<0.0001** |
| **Female, ICM 2, Day 6** | **Female, ICM 1, Day 5** | **0.66** | **0.02** | **<0.0001** |
| Male, ICM 1, Day 6 | Female, ICM 2, Day 5 | 1.23 | 0.09 | 0.0066 |
| Male, ICM 2, Day 6 | Female, ICM 2, Day 5 | 1.00 | 0.07 | 0.4823 |
| **Female, ICM 1, Day 6** | **Female, ICM 2, Day 5** | **1.36** | **0.09** | **<0.0001** |
| Female, ICM 2, Day 6 | Female, ICM 2, Day 5 | 0.97 | 0.04 | 0.2316 |
| **Male, ICM 2, Day 6** | **Male, ICM 1, Day 6** | **0.81** | **0.01** | **<0.0001** |
| **Female, ICM 1, Day 6** | **Male, ICM 1, Day 6** | **1.11** | **0.02** | **<0.0001** |
| **Female, ICM 2, Day 6** | **Male, ICM 1, Day 6** | **0.79** | **0.03** | **<0.0001** |
| **Female, ICM 1, Day 6** | **Male, ICM 2, Day 6** | **1.37** | **0.07** | **<0.0001** |
| Female, ICM 2, Day 6 | Male, ICM 2, Day 6 | 0.97 | 0.03 | 0.2263 |
| **Female, ICM 2, Day 6** | **Female, ICM 1, Day 6** | **0.71** | **0.01** | **<0.0001** |
| p<0.0018 is considered significant (Bonferroni adjusted) and has been bolded | | | | |
